# Prevalence of HBV and HBV vaccination coverage in health care workers of tertiary hospitals of Peshawar, Pakistan

**DOI:** 10.1186/1743-422X-8-275

**Published:** 2011-06-06

**Authors:** Sobia Attaullah, Sanaullah Khan, Naseemullah, Sultan Ayaz, Shahid Niaz Khan, Ijaz Ali, Naseruddin Hoti, Sami Siraj

**Affiliations:** 1 Department of Zoology, Islamia College Peshawar (A Public Sector University) Khyber Pakhtunkhwa, Pakistan; 2 Department of Zoology, Kohat University of Science and Technology Kohat, Pakistan; 3 Lady Reading Hospital Peshawar, Khyber Pakhtunkhwa, Pakistan; 4 Institute of Biotechnology and Genetic Engineering, KP University of Agriculture Peshawar Pakistan; 5 Brady Urological Institute, Johns Hopkins University School of Medicine, Baltimore, MD 21287-2101, USA; 6 Institute of Basic Medical Sciences, Khyber Medical University Peshawar Pakistan

## Abstract

**Background:**

Hepatitis B Virus (HBV) may progress to serious consequences and increase dramatically beyond endemic dimensions that transmits to or from health care workers (HCWs) during routine investigation in their work places. Basic aim of this study was to canvass the safety of HCWs and determine the prevalence of HBV and its possible association with occupational and non-occupational risk factors. Hepatitis B vaccination coverage level and main barriers to vaccination were also taken in account.

**Results:**

A total of 824 health care workers were randomly selected from three major hospitals of Peshawar, Khyber Pakhtunkhwa. Blood samples were analyzed in Department of Zoology, Kohat University of Science and Technology Kohat, and relevant information was obtained by means of preset questionnaire. HCWs in the studied hospitals showed 2.18% prevalence of positive HBV. Nurses and technicians were more prone to occupational exposure and to HBV infection. There was significant difference between vaccinated and non-vaccinated HCWs as well as between the doctors and all other categories. Barriers to complete vaccination, in spite of good knowledge of subjects in this regard were work pressure (39.8%), negligence (38.8%) un-affordability (20.9%), and unavailability (0.5%).

**Conclusions:**

Special preventive measures (universal precaution and vaccination), which are fundamental way to protect HCW against HBV infection should be adopted.

## Background

Among the blood borne pathogens, hepatitis B virus (HBV) has gained the status of global public health threat by being the 10^th ^major deaths causing disease. HBV infects more than 2 billion peoples worldwide, of which over 350 million peoples are chronic carrier [1]. Currently HBV is the leading issue of concern in society and medicine particularly in our under-resourced health care system which lacks the safety measures necessary to avert the risks of infection [2,3]. Different country wide hospital based and population based HBV surveys (individual researchers) estimated a prevalence rate of 2-7%, which places Pakistan in intermediate HBV prevalence zone. During the past two decades this risk has become even more significant as the prevalence of HBV has increased significantly [4], and risk of contracting hepatitis B by HCWs is four fold higher as compared to general adult population [1,5,6].

Worldwide annual proportion of HCWs exposed to HBV infection were about 5.9% [1,5]. In developing countries, 40-60% of HBV infection in HCWs was attributed to professional hazard while in developed countries the attributed fraction was less than 10% due to vaccination coverage [1]. Being the instruments of healthcare system, their interaction with patients is likely to pose unavoidable safety risks for the HCW's [5-7]. The chances of contracting HBV after an HBV-contaminated accidental needlestick average one in 20 while chance of contracting HCV after an accidental needlestick is 3.5 in 100 [4]. In addition to occupational hazards, evidences suggested that certain other unidentified community risk factors put HCWs at risk of acquiring HBV, as the general population. There is a complex multiplicity of risks factors and it is difficult to tease out the biggest contributors to infection in HCWs [8,9].

Hepatitis B is a vaccine preventable disease, though its implementation is still insufficient and a sizeable proportion of HCWs never get vaccinated. The risk of HBV infection in an unvaccinated person from a single HBV-infected needle stick injury ranges from 6-30% [5]. Considering the importance of health care personnel and lack of any significant report in HCWs from the region of Peshawar, this study was designed to investigate the prevalence of HBV in different occupational groups of HCWs, predominant occupational and non-occupational modes of transmission, HBV vaccination coverage level and main barriers to vaccination.

## Methods

### Study location

This study was carried out at three large and tertiary hospitals of Peshawar, Khyber Pakhtunkhwa, namely Khyber Teaching Hospital, Lady Reading Hospital and Hayatabad Medical Complex Hospital, which provides services to the general population all over the province.

### Study sample

The study undertook randomly selected 824 HCWs, working in different departments of the hospitals.

### Inclusion criteria

HCWs with more than 6 months of job experience were included in this study.

### Instrument

The information collection from HCWs was done via interview and through questionnaire. Data set included demographic parameters (gender, marital status, age, duration of employment, education, occupation and working department), history of frequency and nature of activities involved in occupational exposures, history of non-occupational risk factors (blood transfusion, hospitalization, surgery, dental treatment and intravenous drug use), history of HBV vaccination coverage status and time since vaccination, and barriers against vaccination status.

### Laboratory Technique

Blood samples were collected from the population under investigation and tested in Molecular Parasitology and Virology Laboratory, Department of Zoology, Kohat University of Science and Technology (KUST) Kohat, for HBs Ag by immunochromatographic test (ICT). HBV DNA was isolated with GF-1 nucleic acid isolation Kit (Vivantus, USA), S gene of HBV was amplified with nested primers by PCR and amplified DNA was subjected to electrophoresis. The study was conducted after receiving ethical approval from medical superintendents of the respective hospital.

Participated HCWs were grouped into five categories on the basis of their jobs into medical staff including surgeons, physicians and dentists, nursing and midwifery staff, specialized technicians including X-rays, anesthesia and laboratory staff, assistant and general servicing staff including radiology, operational services, attendants, cleaning, laundry, blood bank, pharmacists, dispensers and administrative staff.

### Statistical analysis

Categorical variables were shown in percentages. Chi-square test or Fishers' Exact Test were used to evaluate the association between seropositivity for HBV and categorical variables. A *p *value less than 0.05 was set as significant level.

## Results

Among the selected 824 subjects, 59.8% were male and 40.2% were female, 82.2% were married and 17.8% were unmarried, age ranged from 20-59 years (33.8 ± 8.2 years) and duration of employment from > 6 months to 29 years (14.4 ± 3.21 years). Of the total, 100 HCWs had history of dental treatment, 98 had history of hospitalization, 31 had surgery in the past, 24 had been transfused and only one was intravenous drug user.

**Figure 1:**
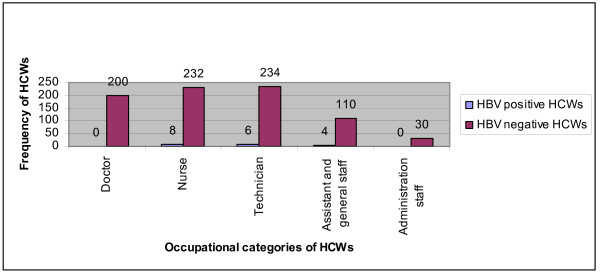
**Distribution of HBV positive and HBV negative HCWs according to occupational categories**.

18 (2.18%) HCWs were found positive for HBV while HBV and HCV mixed infection was not detected in any HCW. Characteristics and distribution (%) of HBV positive and HBV negative are summarized in Table 1. Frequency of HBV-infection in nurses was 44.4%, 33.4% in technicians, 22.3% in assistant staff while no doctor and administrative staff was found positive. Significant association (*P *= 0.328) between HBV prevalence and five occupational categories were present (Figure 1).

**Table 1:** HCWs and HBV positivity

Variables	HBV Negative N (%)	HBV Positive N (%)	Total
Male	483 (59.9)	10 (55.55)	493

Female	323(40.1)	8 (44.44)	331

Married	665(82.5)	12 (66.66)	667

Unmarried	141(17.4)	6 (33.33)	147

Graduate	554(68.7)	1 (5.55)	555

Undergraduate	252(31.3)	17 (94.4)	269

H/O blood transfusion	20(2.48)	4 (22.22)	24

H/O dental treatment	94(11.66)	6(33.33)	100

H/O surgery	27(3.35)	4 (22.22)	31

H/O hospitalization	91(11.2)	7 (38.9)	98

H/O of intravenous drug use	1(0.12)	0 (0)	1

H/O = History of

As for personal data, neither gender (*P *= 1.000), nor marital status (*P *= 0.8575) were found responsible for any statistically significant differences in between HBV negative and HBV positive HCWs. However, a strong correlation was found with age (*P *= 0.0896), employment duration (*P *= 0.0001), dental treatment (*P *= 0.0155), hospitalization (*P *= 0.0013), surgery (*P *= 0.0004) and blood transfusion (*P *= 0.0001). All HCWs were aware of the importance of screening, but overall 24.5% HCWs were aware of their viral status and only four (22.3%) were aware of their seropositivity.

Overall, 572 HCWs were involved in a total of 729 occupational exposures during the past one year period service. Percentage breakdown was 47.3% in nurses, 31.3% in technicians, 13.1% in doctors and 8.2% in assistant staff. Regarding the activity involved in occupational exposure, drawing blood was the main contributor (i.e. 33.6%), followed by recapping of syringes (17%), disposal of used needles (13.3%), insertion of drip (12.6%), trash collection (9.7%), surgical procedure (4.3%), collision with sharp objects (4.1%), fluid splash (3.0%), cleaning instruments (1.8%) and others (0.5%) (Table 2).

**Table 2:** Analysis of risk factors of occupational exposure in HCWs

Activities involved in exposure	Doctor N = 72	Nurse N = 238	Technician N = 209	Assist. & general staff N = 53	Total%	
Taking blood	42	152	32	19	245	33.6

Recapping	6	50	49	19	124	17

Disposal of syringes	12	48	35	2	97	13.3

Insertion of drip	3	47	40	2	92	12.6

Trash collection	1	23	35	12	71	9.7

Surgical instrument	18	9	4	0	31	4.3

Collection with sharp instrument	2	7	19	2	30	4.1

Fluid splash	12	7	2	1	12	3

Cleaning of instrument	0	1	9	3	13	1.8

Others	0	1	3	0	4	0.5

In this study, 605 (73.42%) HCWs had completed their vaccination regimen. Of which 83(13.7%) had been vaccinated before entering the hospital, while 522 (86.3%) received their vaccination after starting their job at the hospital. Regarding the vaccination status, highest coverage was found among doctors, followed by administrative staff, general and assistant staff, technicians and nurses. A significant difference in vaccine coverage in vaccination coverage was found between the doctors and all other categories (*P*= 0.0001) (Table 3).

**Table 3:** HCWs (%) and their HBV vaccination status

Vaccination status	Doctor	Nurse	Technician	Assist. & general staff	Admin. staff	Total
Complete	85	65.4	70	75.6	80	73.4

Incomplete	14.5	30.5	28.4	21.8	20	24.4

Continued	0.5	4.1	1.6	2.6	0	2.2

**Figure 2:**
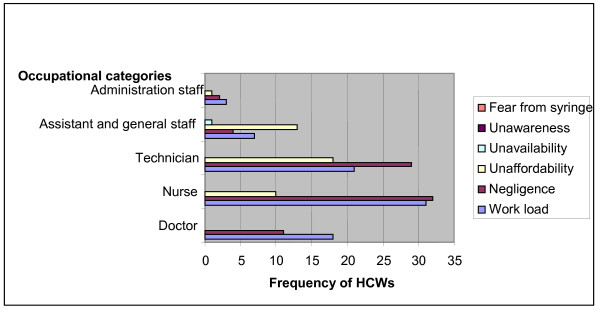
**Analysis of risk factors against HBV vaccination coverage in HCWs**.

The most commonly cited reasons for no vaccination in HCWs found were unaffordability/ high costs (39.8%), work pressure (38.8%), negligence (20.9%) and unavailability (0.5%). Statistical analysis showed significant association between vaccinated verses non vaccinated coverage (*P *= 0.0005) and barriers to complete vaccination (*P*= 0.0001) (Figure 2).

## Discussion

Although the seroprevalence rate of HBV is on the rise in patient's population attending hospitals, screening is not routinely performed in most parts of country because of lack of public awareness, inadequate funding for health care setting and high cost of tests which poses a great threat to the safety of the healthcare workers [10]. The 2.18% prevalence in this study demonstrated that HCWs were at low increased risk for HBV infection when compared with the various previous studies conducted in Pakistan [8-12], except the recent study (0.5%) [2]. The prevalence of HBV varies from place to place, may be due to difference in magnitude of HBV infection in patients, implementation of universal precautions and methodological strategies (employment duration, sample size etc).

In this study age and employment duration strongly correlated with HBV prevalence. One reason may be because seasoned staff member dealt with patients in an inadequately equipped set-up, which predisposed them to these risks even more. In addition to that, there was lack of awareness of personal prophylaxis and probably no or little vaccination option available to them. This logic is also proposed in other literatures [3,7,13-15]. Most of the HBV-infected HCW's in the present study had undergraduate level education. This fact alone is enough to put a rigorous emphasis on their proper education which also is most likely to serve as an effective tool in controlling the professional injuries [15,16]. Nurses and technicians were at high risk of HBV infection as they interact first-hand with the patients [1,5,12,17,18]. Prevalence of HBV might associate with their low socioeconomic status as it is the disease of poverty [3]. Similar to Sarwar and colleagues [9] none of doctor was found positive, but not in accordance to the other studies that among HCWs, surgeons/doctors have the highest risk of HBV infection from their patients [11,19].

Current study showed that excessive risk of exposures among the HCW as a whole as well as among different occupations Nurses had the highest predisposition to occupational accidents, also reported by others [4,6,18,20,21]. Laboratory workers and anesthesia technicians comprised another major portion of occupational injuries, also analysed by the others [4,7,12]. In the current study the percentage of reported incidents in doctors was lower than other studies [4,15,22]. The absolute recording of professional injuries was frequent in nurses but percentage was actually greater in the physicians as this group was found more inclined to self assesses and not report such injuries [22]. The incidence of occupational exposure is common feature of studies across the world [22]. Factors associated with an increased risk of occupational exposure can differ from place to place depending on the standard of facilities [23-25]. Poor economic situation doesn't allow facility and personnel to achieve proper level of sophistication and therein lies the problem [26,27].

Assessment of HBV vaccination coverage in health care setting is needed to evaluate the proportion susceptible to HBV infection [1]. This study demonstrated that vaccination program was successful when compared with previous studies from diverse regions of Pakistan ranging from 37.2% to 66.3% [2,9,12,19,28-30] as well as from other countries and regions such as Africa [1], Nepal [30], Japan, India, North Sydney, South London, Sweden and Egypt [31] but not satisfactory when compared with Australia, New Zealand [1], Iran and UK [30]. In recent study high (81.8%) vaccination status was recorded from Karachi [32] but still universal coverage is not achieved despite the availability of vaccine since 2002 [4]. Most of the western countries recommend the need for immunization against HBV in the start of career in healthcare setting [1] but no such policy is employed in Pakistan, either in letter or in spirit.

It is a matter of fact that the risk factors against the vaccination coverage would vary among different occupations and among different regions of country [33,34]. Collection of the data of the risk factors against the vaccination provides valuable information for identification and evaluation. By eliminating these factors and providing necessary facilities, 100% vaccination coverage is well within the realm of possibility [31]. We believe our study was able to shed light on major hurdles to vaccine coverage, including work pressure, negligence and high cost of the vaccine. We also hope that our study can be used as a precedent to develop important guidelines which, if properly implemented, will be able to curb the one of the root causes of spread of Hepatitis B.

## Authors' contributions

SA and SK designed and gave a critical view of manuscript writing. NU helped in collection of samples/data and SNK performed Lab analysis. IA and SS gave critical view of manuscript writing and participated in data analysis. All the authors' read and approved the final manuscript. SA, NH reviewed the final manuscript and made critical amendments in the data presentation. All authors read and approved the final manuscript.

## Competing interests

The authors declare that they have no competing interests.
